# In-Depth Analysis of Exoproteomes from Marine Bacteria by Shotgun Liquid Chromatography-Tandem Mass Spectrometry: the *Ruegeria pomeroyi* DSS-3 Case-Study

**DOI:** 10.3390/md8082223

**Published:** 2010-07-29

**Authors:** Joseph Alexander Christie-Oleza, Jean Armengaud

**Affiliations:** Laboratoire de Biochimie des Systèmes Perturbés, CEA Marcoule, DSV, iBEB, SBTN, LBSP, F-30207 BAGNOLS-SUR-CEZE, France; E-Mail: joseph.christie@cea.fr (J.A.C.-O.)

**Keywords:** environmental proteomics, exoproteome, marine bacteria, proteogenomics, toxins

## Abstract

Microorganisms secrete into their extracellular environment numerous compounds that are required for their survival. Many of these compounds could be of great interest for biotechnology applications and their genes used in synthetic biology design. The secreted proteins and the components of the translocation systems themselves can be scrutinized in-depth by the most recent proteomic tools. While the secretomes of pathogens are well-documented, those of non-pathogens remain largely to be established. Here, we present the analysis of the exoproteome from the marine bacterium *Ruegeria pomeroyi* DSS-3 grown in standard laboratory conditions. We used a shotgun approach consisting of trypsin digestion of the exoproteome, and identification of the resulting peptides by liquid chromatography coupled to tandem mass spectrometry. Three different proteins that have domains homologous to those observed in RTX toxins were uncovered and were semi-quantified as the most abundantly secreted proteins. One of these proteins clearly stands out from the catalogue, representing over half of the total exoproteome. We also listed many soluble proteins related to ABC and TRAP transporters implied in the uptake of nutrients. The *Ruegeria pomeroyi* DSS-3 case-study illustrates the power of the shotgun nano-LC-MS/MS strategy to decipher the exoproteome from marine bacteria and to contribute to environmental proteomics.

## 1. Introduction

Marine microorganisms have defined the chemistry of the oceans and atmosphere, and represent a large proportion of the biological diversity on Earth. Recent advances in molecular biology techniques changed our perception of microbial diversity [1,2]. Marine microorganisms represent a huge reservoir of enzymatic items that could be useful bricks for the green chemistry field to produce novel biotechnological products of interest. The *Roseobacter* lineage of the class *Alphaproteobacteria* is one of the major bacterial groups in marine environments [3]. They account for up to 25% of marine bacterioplankton communities [4]. *Ruegeria pomeroyi* DSS-3, previously named *Silicibacter pomeroyi* DSS-3 [5], was isolated from marine waters as capable of degrading dimethylsulfoniopropionate [6]. Its genome was sequenced soon after [7]. *R. pomeroyi* DSS-3 is thus the first roseobacterium with a sequenced genome and has served as a model organism for studying the ecophysiological strategies of heterotrophic marine bacteria [7–9]. Its genome analysis defined *R. pomeroyi* DSS-3 as a moderate copiotroph. The abundance of ABC transport genes was discussed and could function to facilitate the capture of a diverse range of low concentration substrates, including those that arise from phytoplankton blooms [9].

All prokaryotic and eukaryotic cells need to exchange compounds with their environment. This does not end with the uptake of nutrients and secretion of cell waste, since many compounds that cells secrete intend to exert an influence on and modify their environment, *i.e.*, antibiotics, toxins, virulence factors, catabolic enzymes, pheromones, and quorum-sensing molecules [10,11]. It is important to differentiate the cellular secretome, which includes all metabolites secreted, extracellular surface molecules and their related transport systems, from the cellular exoproteome, which consists of all proteins found in the extracellular milieu—actively secreted or not [12]. Exoproteomes from several pathogenic bacteria have been described such as the plant pathogen *Erwinia chrysanthemi* [13], the human pathogens *Bacillus cereus* [14,15] and *Streptococcus aureus* [16]. However, the exoproteomes of many bacteria remain unknown, despite the high interest some of the secreted proteins could have for biotechnological use. In gram-positive bacteria, the terms protein secretion and export are synonyms due to their single monoderm membrane. On the other hand, in gram-negative bacteria, protein secretion makes reference to those systems that transport proteins though the lipid bilayer directly to the bacterial milieu, whereas protein export is used for those proteins transported to the periplasm [12]. Eight translocation systems though the cytoplasmic membrane and eight though the outer membrane have been described in gram-negative bacteria (reviewed in [17]). Three of the former translocation systems (type I, III and IV) can carry out the secretion of proteins directly though the gram-negative bilayer. Protein secretion can also be carried out in a two-step translocation in type II secretory pathway. Several algorithms have been developed to predict the presence of signal peptides with the protein sequence as input [18]. The SignalP predictor [19] achieves the best overall accuracy in benchmarking experiments and signal peptide predictions, and usually corresponds well with what is observed experimentally, as shown by several proteomic studies [20–22]. However, signal peptide detection does not ensure protein secretion to the exoproteome, as the protein can be simply exported to the periplasm or stay anchored to the membrane [12]. Also, proteins with no signal peptide are not considered to be secreted in the *in silico* approaches, although they can be found experimentally in the exoproteome. Therefore, protein analysis considering integrative aspects should be taken into account. In this respect, the LocateP tool, developed for gram-positive bacteria, is an interesting predictor [23].

With the concomitant development of novel mass spectrometry ionization modes, improvement of mass analyzers, and release of numerous complete genome sequences, proteomics has become a successful approach in biology. Applications of proteomics in microbial biotechnology and environmental proteomics are already well documented [24–26]. After a period centered on two-dimensional (2D)-gel electrophoresis separation of proteins and mass fingerprint, larger scale approaches to study complex mixture of proteins are now feasible with the use of high-throughput liquid chromatography-tandem mass spectrometry (LC-MS/MS) advanced technology [27]. Logically, bacteria exoproteomes have been first investigated through time-consuming 2D-gel approaches [28,29], but nano-LC-MS/MS approaches are now more frequently used for a more comprehensive analysis [14,30,31].

To date, the exoproteome analysis of *Pseudoalteromonas tunicata* is the only example illustrating a marine bacterium exoproteome [32]. It pointed to the importance of iron transport and acquisition. To further explore the exoproteome of other marine bacteria, we chose the *Roseobacter* strain *R. pomeroyi* DSS-3 as a model because of the presence of numerous virulence genes that has been noted in this genome [7]. Here, we present a reliable method to carry out a fast analysis and semi-quantification of the exoproteome of marine bacteria. Data recorded for *R. pomeroyi* DSS-3 shows a low cytoplasmic contamination and an elevated number of ABC and TRAP related transporters, in agreement with *R. pomeroyi*’s lifestyle. Three proteins far-related to RTX-like toxins were detected as the most abundant proteins, one of them representing over 50% of the total exoproteome.

## 2. Results and Discussion

### 2.1. A shotgun nano-LC-MS/MS strategy for a comprehensive exoproteome

**Figure 1:**
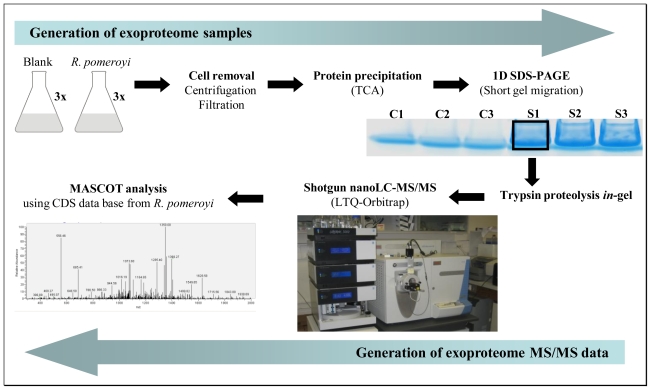
Schematic flow describing the nano-LC-MS/MS shotgun strategy used for *R. pomeroyi* exoproteome analysis. Culture supernatant production, protein extraction, proteolysis and nano-LC-MS/MS analysis are shown.

To investigate the exoproteome of *R. pomeroyi* DSS-3, we setup a novel shotgun proteomic approach that could quickly identify and semi-quantify the major proteins found in the extracellular medium for a given growth condition. Figure 1 shows how the samples were generated and processed. First, three independent liquid cultures of *R. pomeroyi* DSS-3 were carried out in marine broth. Cell growth was stopped during the exponential phase (when OD_600_ reached 0.6) to avoid excessive cell death and subsequent cytoplasmic spill into the medium. The supernatant of each of these cultures was collected after centrifugation. Precautions were taken during culture centrifugation and supernatant filtering to avoid cell damage. In parallel, three flasks with the same sterile medium were launched without bacterial inoculum. They were incubated and processed in the same way to obtain experimental controls. Analysis of these controls is important due to high amounts of large protein fragments and peptides present in peptone and yeast extract, two main components of the marine broth. Proteins from the six filtered supernatants were concentrated by precipitation with trichloroacetic acid (Figure 1). The resulting samples were then dissolved into LDS buffer and loaded on a SDS-PAGE gel that was run for a short migration time only. This allows removing small molecules from the samples and cleaning denatured polypeptides trapped in the polyacrylamide gel. Each sample was excised from the gel as a 3 × 4 mm^2^ band. Trypsin proteolysis was carried out *in*-gel. The resulting complex peptide mixture was extracted and then injected onto a C18 reverse phase chromatography column coupled to a high resolution LTQ-Orbitrap hybrid mass spectrometer (Figure 1). Tandem mass spectra were acquired and interpreted with the complete CDS database of *R. pomeroyi*. Analysis of the three control samples only detected bovine trypsin autolytic peptides and a few peptides signing the presence of human keratins. As these peptides are common proteomic contaminants, we implemented the corresponding protein sequences in the database for subsequent searches and systematically removed the corresponding assigned MS/MS spectra.

### 2.2. A broad exoproteome is revealed in a one-shot analysis

A total of 15,261 MS/MS spectra were detected when considering the three biological repeats. Among this impressive dataset, 4,233 MS/MS spectra were assigned to peptides belonging to *R. pomeroyi* proteins. A total of 469 different tryptic peptides were listed (Supplementary Data, Table S1), most of them being detected several times. These peptides allowed the identification of 60 polypeptides when applying the consensual rule in proteomics of at least two detected peptides required for validating the presence of a given polypeptide in a sample. Supplementary Data Table S2 lists the characteristics of these proteins from *R. pomeroyi*.

**Figure 2:**
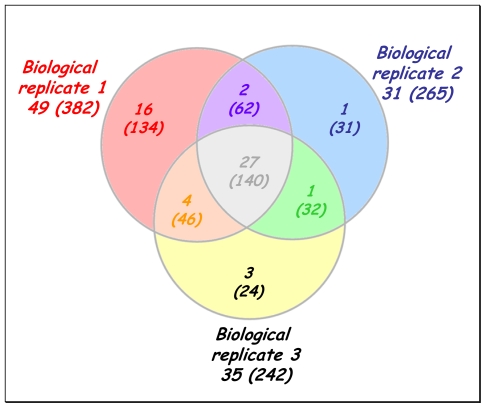
Venn diagram comparing the number of identified proteins and peptides for the three biological replicates. The numbers of proteins detected with at least two peptides and the total number of peptides validated with a p value below 0.005 are indicated by the numbers without and within brackets, respectively.

Figure 2 shows a Venn diagram reporting the number of different proteins and the corresponding peptides detected per biological replicate. From this diagram, it can be seen that the three biological replicates showed a quite similar exoproteome pattern. In 2004, Liu *et al*. reported a label-free semi-quantitative method based on MS/MS spectral counts [33]. These authors demonstrated that for a given protein in a mixture, its MS/MS spectral count correlates linearly with the protein abundance within over two orders of magnitude. Since then, numerous semi-quantitative studies based on this concept have been reported [34]. Supplementary Data Table S2 compiles the number of MS/MS spectra that were detected for each polypeptide in the three biological replicates. In this study, the three most detected proteins based on the total MS/MS spectra recorded are: YP_165496, YP_165625 and YP_168868.

Table 1 shows the characteristics of the detected proteins in terms of prediction of peptide signal and functional annotation. The three most detected proteins account for 2610 (61%), 359 (8%), and 164 (4%) MS/MS spectra for YP_165496, YP_165625 and YP_168868, respectively. These results clearly indicate that the former is the most abundant protein in the *R. pomeroyi* secretome. It was annotated based on similarity search as a homologue of the PaxA protein (AAF15370) reported in *Pasteurella aerogenes* and found to exhibit a co-hemolytic activity in the presence of the sphingomyelinase of *Staphylococcus aureus* [35]. Due to the high sensitivity of our MS/MS analysis, numerous less abundant proteins were also detected (Supplementary Data, Table S2). Some of these correspond to central metabolism enzymes that should be cytoplasmic as they do not exhibit any predicted signal peptide, such as the elongation factors Tu and Ts, or the succinyl-CoA synthase. Their identification may indicate that some cells could have suffered lysis during culture or during the supernatant collecting process. Anyway, such lysis is probably very infrequent because only a few ribosomal factors were identified in the exoproteome although quite abundant inside the cells. We can observe among the exoproteome list many proteins which have a predicted signal peptide for secretion, and are thus logically expected to be part of the *R. pomeroyi* secretome. As indicated in Table 1, a majority of polypeptides amongst these are ABC and TRAP transporters.

### 2.3. Confirmation of the nature and over-representation of the most abundant proteins

**Figure 3:**
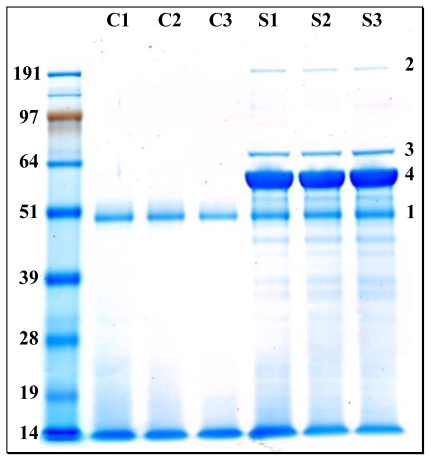
Exoproteomes from *R. pomeroyi* DSS-3 and controls resolved by SDS-PAGE. Exoproteome samples from the control flasks (wells labeled C) and R. pomeroyi DSS-3 cultures (wells labeled S) were run on a 10% SDS-PAGE gel for a long migration for full band resolution. The gel was stained with SimplyBlue SafeStain (Invitrogen) and destained overnight with ultrapure water. The corresponding molecular weights of each protein marker (SeeBlue Plus2 marker from Invitrogen) are indicated in kDa. Numbers correspond to relevant bands commented in the text (band 1: putative protease used for peptone digestion; band 2: YP_168868; band 3: YP_165625 and band 4: YP_165496).

In order to confirm the relative quantities of the main proteins found in the exoproteome, we reloaded the samples onto a SDS-PAGE gel, but this time the proteins were fully resolved. Figure 3 shows the corresponding gel that was stained with Coomassie blue. As expected, control cultures revealed a low content in polypeptides with a low molecular weight smear corresponding to the enzymatically digested proteins from the peptone. The intense diffuse band seen in all samples (labeled band 1 in Figure 3) probably corresponds to the protease used in the peptone digestion. All three samples of *R. pomeroyi* exoproteome consistently presented identical well defined bands in addition to what was observed in the control samples. Clearly, one major protein (band 3) stands out among the rest and should correspond to the YP_165496 polypeptide highly detected in the shotgun approach. However, from the gel shown Figure 3, the molecular weight of this protein appears to be 6–8 kDa higher than theoretically expected when taking its sequence into consideration. We evaluated by densitometry that this protein stands for over 53% of the total protein extract. We confirmed the identity of this protein as YP_165496 by an *in*-gel proteolysis of the corresponding band and MS/MS identification of the resulting trypsic peptides (Supplementary Data, Table S3). We verified that its sequence coverage with the detected peptides was slightly lower than observed in the shotgun approach, *i.e.*, 54%. The two other major proteins detected in the shotgun approach, YP_165625 and YP_168868, should have a theoretical molecular weight of 76.1 and 225.0 kDa, respectively. These proteins are readily observed in the gel shown in Figure 3 (band 2 and band 1, respectively). The densitometry analysis revealed that these bands correspond to 6% and 1% of total protein extract, respectively. We also confirmed their two assignments by MS/MS analysis (Supplementary Data, Table S3).

### 2.4. Structural and functional hints regarding YP_165496

**Figure 4:**
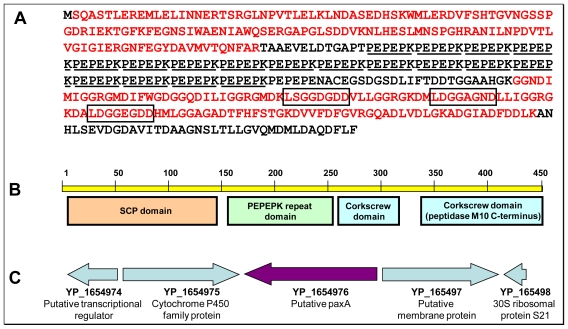
Characteristics of YP_165496, the most abundant protein in the *R. pomeroyi* DSS-3 exoproteome. (**A**) Amino acid sequence of the hitherto annotated YP_165496. Peptides identified by MS/MS are shown in red. The 18 PEPEPK motif repeats are underlined. The three conserved glycine-rich sequences typically seen in the *C-*terminal peptidase-like domains of the RTX-like hemolytic toxins (L/I/F-X-G-G-X-G-N/D-X) are highlighted with squares; (**B**) Conserved domains detected in the YP_165496 protein. The numbering indicates the amino acid position in the polypeptide sequence; (**C**) Genomic context of the YP_165496 encoding gene on the chromosome of *R. pomeroyi* DSS-3. Genes are schematized with arrows. Names and annotation of the corresponding proteins are indicated.

A total of 45 different peptides assigned to the YP_165496 protein have been identified. Figure 4 (Panel A) shows the sequence of the protein as presently annotated and its peptide coverage. The peptides detected in our study cover over 58% of the predicted protein sequence. It is worth to note that these peptides cover very well the *N-*terminus and *C-*terminus of the polypeptide. However, the central region is not at all covered. In this 151 amino acid region we found a perfectly repeated (×18) motif of six amino acids: PEPEPK. This region is highly charged with 18 basic residues and 39 acidic residues. The presence of a lysine at the end of this motif generates after trypsin proteolysis a peptide too short for detection. On the other hand, the presence of a proline at the beginning of the motif could inhibit only partially proteolysis [36]. As mentioned here above, we were surprised to see this polypeptide migrating as a 56 kDa protein in SDS-PAGE while it is predicted to exhibit a molecular weight of 48.8 kDa. The denaturing conditions with SDS and β-mercaptoethanol prevent the possibility of heteromeric quaternary structure and disulfide bonds, respectively. In addition, we did not detect the presence of another plausible associated protein sequence mixed with YP_165496 when analysing peptide digest from band 4. In order to explain the unexpected size of the YP_165496 estimated experimentally, we searched our MS/MS data set with specific proteogenomic approaches. As previously described [37], proteogenomic searches using a database comprising all possible ORFs from the six-frame whole genome translation are useful in determining the exact protein translational start codon or the presence of an unexpected translational frameshift. First, no peptides were detected in the present study upstream from the presently annotated start methionine of YP_165496. Second, no peptides matched any of the possible ORFs downstream from YP_165496. Moreover, the most *N-*terminal peptide found in our study is the [SQASTLER] peptide. Indeed, this peptide confirms the current translational start that results in the production of a polypeptide starting with the sequence MSQASTLER. The protein is then matured after deformylation with removal of the initial methionine by the methionine aminopeptidase found in all organisms [38]. This maturation was predicted as the second amino acid (here, a serine) is a small lateral chain residue [39,40]. We thus concluded that the unexpected migration observed for YP_165496 in SDS-PAGE could probably result from the unusually highly charged region made of the 18 motif repeats.

PSI-BLAST analysis revealed that the closest relative to the YP_165496 protein is RBY4I_276 (ZP_05077090), a protein from *Rhodobacterales bacterium Y4I* that contains a well conserved *N-*terminus (74% identity) and a less conserved *C-*terminus (46% identity) than those found in YP_165496. Interestingly, no hexa-amino acid motif repeated region was found in RBY4I_276. Figure 4 shows the four domains that we detected in YP_165496: a SCP-like domain at the *N-*terminus, the PEPEPK motif repeated region, and two peptidase M10 serralysin-like *C-*terminal domains at the *C-*terminus. The SCP domain is found in various proteins annotated as extracellular but its biological role is unknown. In mammals, such domains are found in proteins involved in several important developmental processes [41]. They are also commonly used by insects and reptiles as mammalian toxins (allergen 5 from vespid venom for example), and are essential in plants, being annotated as pathogenesis-related proteins. The *C-*terminal domain resembles those found in serralysins, peptidases related to mammalian matrix metallopeptidases. While the peptidase active site is found in the *N-*terminus of these serralysins, the *C-*terminal domain forms a corkscrew and is thought to be important for secretion of the protein through the bacterial cell wall. Such a *C-*terminal domain has been found in the PaxA protein from *Pasteurella aerogenes* [35]. PaxA proteins are described as RTX-like hemolytic toxins and are known to have repeated glycine-rich sequences L/I/F-X-G-G-X-G-N/D-X conserved in the *C-*terminal and peptidase-like domains. In the sequence of the YP_165496 protein detected from the exoproteome of *R. pomeroyi*, we found three of these repeats separated by 10 amino acids (Figure 4). RTX toxins are commonly seen in gram negative bacteria and, although soluble in aqueous solutions, are able to insert into the host cell membrane carrying out its lysis effect [42]. They seem to have host specificity, being in all cases described in pathogenic species [43–45]. RTX genes are classically organized in operons comprising the activator gene *paxC* upstream of the structural toxin gene *paxA*, which is followed by the secretion protein genes *paxB* and *paxD*. Although YP_165496 shares the same *C-*terminal corkscrew domain as PaxA from *P. aerogenes*, its annotation as a PaxA homologue in *R. pomeroyi* is far to be trivial. The analysis of the close neighborhood of its structural gene onto the chromosome (Figure 4) did not reveal any operonic structure as seen for RTX-like hemolytic toxins [35,44]. In any case, YP_165496 is full-length homologous to RTX toxins and possesses the atypical PEPEPK repeated domain.

### 2.5. Structure and role of the two other major secreted proteins

The other two major detected proteins in the *R. pomeroyi* exoproteome, YP_165625 and YP_168868, are both annotated as type I secretion target repeat-containing proteins. Like YP_165496, they present similarities with the peptidase M10 serralysin-like *C-*terminal domain found in the *C-*terminus of RTX-like toxins. They both presented the RTX glycine-rich repeat units at the *C-*terminal end of the protein sequence together with the peptidase domains. Like in the case of YP_165496, no signal peptide was detected in these polypeptides indicating an alternative non-conventional secretion system. By PSI-BLAST searches, we found that the *N-*terminal domain of both proteins is not at all conserved among the bacterial kingdom. These two proteins are thus quite specific of *R. pomeroyi.*

With a predicted sequence of 719 amino acids, YP_165625 was extensively covered by MS/MS peptide detection with over 86% of the total sequence covered (taking into account the three shotgun and the band analysis). This included both *N-* and *C-*termini, indicating that this protein plausibly does not undergo post-translational splicing for activation or transport. The closest homologue to the proteins *C-*terminal domain is another protein encoded in the *R. pomeroyi* genome (YP_164986), although this giant protein of 1478 residues is not at all detected in our shotgun proteomic data. The YP_165625 protein as a whole has a homologue in another alpha-proteobacterium, *Roseobacter* sp. SK209-2-6 (ZP_01755840). These two proteins share 29% of identities over their full-length sequences.

YP_168868 is a larger protein of 2,164 amino acids, having MS/MS peptides detected in the shotgun analysis homogeneously found throughout the sequence and covering 34% of the protein. Seven repeats of the corkscrew domain found in RTX-toxins and YP_165496 are detected in the *C-*terminus of this polypeptide. Noteworthy, no peptides matching the region between amino acid positions 149 and 372 were detected. This finding could be explained by the presence of a predicted intein domain in this region. Intein sequences, using an autocatalytic system, are able to excise from the flanking exteins in the polypeptide sequence for dispersion, being the flanking regions joint by a peptide bond [46,47]. In this case we find at position 158 the *N-*terminal splicing region (KVSIVEDKLEIAAEKLGLNSTTLSDAINNCFVAG) separated by 148 amino acids, region where the endonuclease or linker domain is homed, from the *C-*terminal splicing region (YNFEVEDFHTYIADGNRVHN), ending at position 359.

Analysis of the genomic context of their structural genes in *R. pomeroyi* genome revealed YP_165625 coding gene is flanked by a succinate dehydrogenase operon and plausible urea decarboxylase genes while YP_168868 is flanked by a copper oxidase domain protein and an IS4-like transposase. It is difficult at this point to establish a functional relationship between these two proteins and YP_165496, although we do not discard the possibility of a cooperational effect of all three plausible toxins to eliminate environmental competitors. The fact that marine bacteria [48] and, more precisely, *R. pomeroyi* [49] harbor genes coding for potential toxins or virulent factors is not new. But it is the first time potential toxins have been demonstrated by proteomics to be synthesized and secreted in a marine bacterium.

### 2.6. Exoproteins related to ABC and TRAP transporters

A high number of soluble proteins found in the exoproteome are related to ABC and TRAP transporters implied in the uptake of diverse compounds. Such a result is not surprising because *R. pomeroyi* has an unusually high number of these transporters encoded in its genome. They should be crucial to help *R. pomeroyi* to cope with a nutrient-poor ocean [7]. As listed in Table 1, up to 13 of the 16 proteins related to such transporters and found in the exoproteome have a high probability to comprise a signal peptide. The major part of these substrate-binding proteins (8 of the 16 detected) are related to the uptake of peptides or amino acids, and this may be due to the growth of *R. pomeroyi* in a rich peptide media. These exoproteins mainly have two cell-membrane translocation functions: binding a specific substrate, and interacting with the membrane bound complex. As found by PSI-BLAST searches, the 16 proteins have close homologues in other *Roseobacter* strains and these homologues are all annotated as transport-related proteins. However, their sequence alignments show that their *N-*termini are not correctly identified in some strains. This still happens frequently in genome annotations as recently shown for the *Deinococcus deserti* bacterium by means of a specific chemical labelling of protein *N-*termini [50]. Here, we did not predict the presence of a signal peptide in three proteins (labelled with an asterisk in Table 1). Interestingly, these three proteins do not exhibit a correct BLAST alignment with their homologues at their *N-*termini. Their signal peptides could have been missed in terms of annotation. The three proteins YP_168201, YP_165781 and YP_166898 were re-annotated in terms of *N-*terminus with the choice as translational start codon of an internal ATG, based on sequence alignments with their closest homologues. The three proteins are now 31, 42, and 69 amino acids shorter than previously annotated, respectively. They were re-analyzed by the SignalP 3.0 predictor. In this case, a signal peptide was predicted with a high probability in all the three proteins (Supplementary Data, Table S2). This result confirms our updated annotation.

### 2.7. Other proteins identified with a signal peptide

Among the remaining proteins detected in the exoproteome and predicted to encompass a signal peptide we found two hypothetical proteins. Table 1 reports their characteristics. The main objective of proteogenomics is to define the pool of existing proteins and remove dubious ORFs from genome annotations [27]. As we clearly identified these two proteins, the corresponding genes should be re-annotated without the mention “hypothetical”. Here, YP_165456 shares some similarities with tricarboxylic transport proteins related to TRAP transporters, being possibly implied in the uptake of such compounds. The second protein, YP_167503, shares evident similarities with the periplasmic YceI protein (COG2353) from *Escherichia coli* [51]. Although of unknown function, this protein could be related with metabolism, transport or storage of isoprenoid quinones [52].

In addition to these hypothetical proteins, we found two proteins, namely YP_167495 and YP_165589, which are related to fatty acid degradation. These proteins exhibit an acyl carrier protein domain and a thiolase domain, respectively. Both proteins could have a role in facilitating fatty acid uptake by the cell. Moreover, YP_167817 and YP_167786, both annotated as solute-binding family 7 proteins, are extracellular proteins that are related to TRAP transporters although the compound they bind is not clearly defined. The function of the annotated peptidyl-prolyl cis-trans isomerase YP_167459 is to isomerize peptide bonds to accelerate protein folding. As we found this protein in the exoproteome, it could play a role in enabling peptide uptake. The three remaining proteins listed in Table 1 are membrane proteins that could have been released from the cells.

## 3. Experimental

### 3.1. Exoproteome samples

Three flasks containing 40 mL of marine broth (Difco) were inoculated with *Ruegeria pomeroyi* DSS-3 cells (DSM15171T) previously grown on agar plates. The cultures were incubated at 26 °C under 180 rpm agitation. In parallel, three flasks without inoculum were processed in the same way. Cultures were allowed to grow until mid exponential phase (OD_600_ = 0.6) and then centrifuged at 3,000 g for 10 min at 20 °C. Supernatants were carefully filtered through two low protein-binding filters of 0.45 μm (Millex-HV) and then 0.22 μm (Millex-GV) diameter pore (Millipore) to eliminate any remaining cells. Protein concentration and purification was carried out by trichloroacetic acid precipitation. Briefly, the 40 mL filtered supernatants were incubated 10 min with 0.015% (v/v) of deoxycholic acid (Sigma-Aldrich) at room temperature. Trichloroacetic acid (Sigma-Aldrich) was added at a final 3% (v/v) and incubated 30 min on ice. Samples were centrifuged at 3,000 g for 15 min at 4 °C. The resulting pellets were resuspended in 1 mL of ultrapure water. Deoxycholic acid and trichloroacetic acid treatments were repeated. The final pellets were further dissolved into 500 μL of 1:1 ethanol:ether (v/v) and centrifuged at 4 °C for 15 min at 13,000 g. Pellets were dried using a SPD121 SpeedVac (Savant). Finally, pellets were dissolved in 90 μL of lithium dodecyl sulphate-β-mercaptoethanol protein gel sample buffer (Invitrogen) and incubated at 99 °C for 5 min prior SDS-PAGE.

### 3.2. Trypsin in-gel proteolysis and nano-LC-MS/MS analysis

The three blanks and the three *R. pomeroyi* DSS-3 exoproteome samples (20 μL) were loaded on the same 10% Tris-Bis NuPAGE gel (Invitrogen). SDS-PAGE was carried out using 1X 3-(N-morpholino)propanesulfonic acid solution (Invitrogen) as the running buffer. For the one nano-LC-MS/MS run shotgun analysis, proteins were resolved only over a 3 mm short migration. To visualize the fully resolved pattern of exoproteomes, 5 μL of each sample was loaded onto another SDS-PAGE gel and a long migration was carried out. Gels were stained with SimplyBlue SafeStain, a ready-to-use Coomassie G-250 stain from Invitrogen. SeeBlue Plus2 (Invitrogen) was used as the molecular weight marker. Polyacrylamide gel bands (equivalent in volume to 50 μL) were cut and processed for *in*-gel proteolysis with trypsin (Roche) following the ProteasMax protocol (Promega) as previously described [14]. Nano-LC-MS/MS experiments were performed using the LTQ-Orbitrap XL hybrid mass spectrometer (ThermoFisher) coupled to an UltiMate 3000 LC system (Dionex-LC Packings). Conditions used were those previously described [53].

### 3.3. MS/MS database search

The recorded MS/MS spectra were searched against a home-made protein sequence database containing all the annotated CDS sequences (NC_003911 and NC_006569) of the *R. pomeroyi* DSS-3 genome [7]. The search was carried out with the MASCOT 2.2.04 software (Matrix Science). Parameters were established as follows: tryptic peptides with a maximum of 1 miss cleavage during proteolytic digestion, a mass tolerance of 5 ppm on the parent ion and 0.5 Da on the MS/MS, fixed modification for carboxyamidomethylated Cys and variable modification for oxidized Met. MASCOT results were parsed using the IRMa 1.22.4 software [54], filtering peptides with *p* value below 0.005. False-positive rate for peptide identification was estimated using a reverse decoy database as below 0.5% with these parameters. A protein was considered validated when at least two different peptides were detected in the same experiment. False-positive rate for protein identification was estimated using a reverse decoy database as below 0.1% with these parameters.

### 3.4. Protein quantification

Protein abundance was evaluated in the shotgun analysis by MS/MS spectral counts as previously described [55]. Densitometry analysis of the SDS-PAGE gel stained with Coomassie blue was carried out with the Quantity One software from BioRad. Densitometry value of each protein band was the mean ratio obtained by dividing the signal of the band of interest with the total signal of the gel lane for the three biological duplicates. The signal observed in the band and the whole lane in the blank samples was taken into account.

### 3.5. Protein and nucleic sequence analysis

PSI-BLAST and protein domain searches were performed with the NCBI website facilities (blast.ncbi.nlm.nih.gov) using the non-redundant protein sequences database and default parameters. For the prediction of signal peptides, *R. pomeroyi* proteins were analyzed using the SignalP 3.0 server (www.cbs.dtu.dk/services/SignalP/) with settings for Gram-negative bacteria. Protein molecular weights and isoelectric points were calculated using the protparam software from the Expasy toolbox (www.expasy.ch/tools/protparam.html). GBrowse algorithm from the Roseobase website (www.roseobase.org) was used to browse the *Ruegeria pomeroyi* DSS-3 genome.

## 4. Conclusions

This work demonstrates the utility and high reliability of shotgun nano-LC-MS/MS analysis for a quick survey of exoproteomes from marine bacteria. We have shown how the components of a complex protein mixture can be identified, semi-quantified in a relatively simple way, and the most abundantly secreted proteins ranked. Our shotgun approach compares well with traditional proteomic approach based on 2D-gel electrophoresis. In the three nano-LC-MS/MS runs, we identified 60 different proteins in the exoproteome of *R. pomeroyi*, while other recent works on exoproteomes from bacteria report the identification of roughly the same amount of proteins but with larger technical efforts [28]. We also applied the latest trends from the proteogenomics field [27] to re-annotate some genes of *R. pomeroyi* using the protein information derived from the MS/MS data recorded. Exoproteome data are also useful to confirm the predicted secreted proteins and, therefore, the established catalogue of proteins secreted could be helpful for improving secreted prediction tools. More importantly, our results highlight the biotechnological potential of marine bacterial strains. Here, we explored the exoproteome of the non-pathogenic marine bacterium *R. pomeroyi* DSS-3. Besides the many ABC and TRAP-related proteins detected, which are somehow expected for growth of *R. pomeroyi* DSS-3 in a poor nutrient ocean, we found three highly abundant proteins that could be of high biotechnological interest. Surprisingly, one of these proteins represented over half of the total exoproteome, when either estimated by MS/MS spectral counts or evaluated by densitometry after SDS-PAGE resolution of the protein mixture. The biosynthetic cost of secreted proteins was previously shown to be under intense selective pressure, especially for highly expressed proteins [56]. The role of this protein is thus probably really crucial for *R. pomeroyi*. These three proteins comprise domains similar to those observed in RTX toxins. Biochemical tests have to be now performed to further evaluate their function and their biotechnological potential.

## Figures and Tables

**Table 1:** Most detected proteins from *R. pomeroyi* exoproteome.

Accession	Function	# peptides	# MS/MS	Signal Peptide prediction
**Most abundant proteins detected**				
YP_165496	PaxA, putative	45	2610	absence
YP_165625	type I secretion target repeat-containing protein	48	359	absence
YP_168868	type I secretion target repeat-containing protein	37	164	absence
**Proteins ABC and TRAP transporters**				
YP_168968	oligopeptide/dipeptide ABC transporter, periplasmic	13	58	presence
YP_168022	peptide/opine/nickel uptake ABC transporter periplasmic	16	54	presence
YP_168201	peptide/nickel/opine uptake ABC transporter periplasmic	10	42	presence *
YP_165781	glutamate/glutamine/aspartate/asparagine ABC transporter, periplasmic	10	39	presence *
YP_168669	polyamine ABC trasnporter, periplasmic polyamine-binding protein	6	28	presence
YP_165868	sugar ABC transporter, periplasmic sugar-binding protein	7	24	presence
YP_167838	TRAP transporter solute receptor DctP family protein	6	19	presence
YP_167174	branched-chain amino acid ABC transporter, periplasmic	4	14	presence
YP_167412	TRAP transporter solute receptor TAXI family protein	5	13	presence
YP_165067	TRAP dicarboxylate transporter, DctP subunit	4	13	presence
YP_165642	sugar ABC transporter, periplasmic sugar-binding protein	3	12	presence
YP_166898	oligopeptide/dipeptide ABC transporter, periplasmic	2	10	presence *
YP_166382	glycine betaine/proline ABC transporter, periplasmic	2	5	presence
YP_165960	oligopeptide ABC transporter, periplasmic	3	4	presence
YP_166114	xylose ABC transporter, periplasmic xylose-binding protein	2	2	presence
YP_167025	ABC transporter, periplasmic substrate-binding protein	2	2	presence
**Other proteins with predicted signal peptide**				
YP_165456	hypothetical protein SPO0186	10	37	presence
YP_167495	3-oxoacyl-(acyl carrier protein) synthase II	4	21	presence
YP_167459	peptidyl-prolyl cis-trans isomerase, cyclophilin-type	6	20	presence
YP_165903	bmp family protein	4	14	presence
YP_165402	cytochrome c family protein	4	12	presence
YP_165589	acetyl-CoA acetyltransferase	4	9	presence
YP_168626	outer membrane porin	3	7	presence
YP_167817	solute-binding family 7 protein	2	5	presence
YP_167786	solute-binding family 7 protein	3	4	presence
YP_167503	hypothetical protein SPO2279	2	4	presence

* Proteins/genes that have been re-annotated.

## References

[1] Thakur NL Jain R Natalio F Hamer B Thakur AN Muller WE Marine molecular biology: an emerging field of biological sciences Biotechnol. Adv 2008 26 233 245 18299181 10.1016/j.biotechadv.2008.01.001

[2] Yooseph S Sutton G Rusch DB Halpern AL Williamson SJ Remington K Eisen JA Heidelberg KB Manning G Li W The Sorcerer II Global Ocean Sampling expedition: expanding the universe of protein families PLoS Biol 2007 5 e16 17355171 10.1371/journal.pbio.0050016 PMC1821046

[3] Brinkhoff T Giebel HA Simon M Diversity, ecology, and genomics of the Roseobacter clade: a short overview Arch. Microbiol 2008 189 531 539 18253713 10.1007/s00203-008-0353-y

[4] Wagner-Dobler I Biebl H Environmental biology of the marine Roseobacter lineage Annu. Rev. Microbiol 2006 60 255 280 16719716 10.1146/annurev.micro.60.080805.142115

[5] Yi H Lim YW Chun J Taxonomic evaluation of the genera *Ruegeria* and *Silicibacter*: a proposal to transfer the genus *Silicibacter* Petursdottir and Kristjansson 1999 to the genus *Ruegeria* Uchino *et al.* 1999 Int. J. Syst. Evol. Microbiol 2007 57 815 819 17392212 10.1099/ijs.0.64568-0

[6] Gonzalez JM Covert JS Whitman WB Henriksen JR Mayer F Scharf B Schmitt R Buchan A Fuhrman JA Kiene RP Moran MA *Silicibacter pomeroyi* sp. nov. and *Roseovarius nubinhibens* sp. nov., dimethylsulfoniopropionate-demethylating bacteria from marine environments Int. J. Syst. Evol. Microbiol 2003 53 1261 1269 13130004 10.1099/ijs.0.02491-0

[7] Moran MA Buchan A Gonzalez JM Heidelberg JF Whitman WB Kiene RP Henriksen JR King GM Belas R Fuqua C Genome sequence of *Silicibacter pomeroyi* reveals adaptations to the marine environment Nature 2004 432 910 913 15602564 10.1038/nature03170

[8] Burgmann H Howard EC Ye W Sun F Sun S Napierala S Moran MA Transcriptional response of *Silicibacter pomeroyi* DSS-3 to dimethylsulfoniopropionate (DMSP) Environ. Microbiol 2007 9 2742 2755 17922758 10.1111/j.1462-2920.2007.01386.x

[9] Lauro FM McDougald D Thomas T Williams TJ Egan S Rice S DeMaere MZ Ting L Ertan H Johnson J Ferriera S Lapidus A Anderson I Kyrpides N Munk AC Detter C Han CS Brown MV Robb FT Kjelleberg S Cavicchioli R The genomic basis of trophic strategy in marine bacteria Proc. Natl. Acad. Sci. USA 2009 106 15527 15533 19805210 10.1073/pnas.0903507106 PMC2739866

[10] Antelmann H Tjalsma H Voigt B Ohlmeier S Bron S van Dijl JM Hecker M A proteomic view on genome-based signal peptide predictions Genome Res 2001 11 1484 1502 11544192 10.1101/gr.182801

[11] Preston GM Studholme DJ Caldelari I Profiling the secretomes of plant pathogenic Proteobacteria FEMS Microbiol. Rev 2005 29 331 360 15808747 10.1016/j.femsre.2004.12.004

[12] Desvaux M Hébraud M Talon R Henderson IR Secretion and subcellular localizations of bacterial proteins: a semantic awareness issue Trends Microbiol 2009 17 139 145 19299134 10.1016/j.tim.2009.01.004

[13] Kazemi-Pour N Condemine G Hugouvieux-Cotte-Pattat N The secretome of the plant pathogenic bacterium Erwinia chrysanthemi Proteomics 2004 4 3177 3186 15378709 10.1002/pmic.200300814

[14] Clair G Roussi S Armengaud J Duport C Expanding the known repertoire of virulence factors produced by Bacillus cereus through early secretome profiling in three redox conditions Mol. Cell. Proteomics 2010 10.1074/mcp.M000027-MCP201 PMC2938089 20368289

[15] Gohar M Gilois N Graveline R Garreau C Sanchis V Lereclus D A comparative study of Bacillus cereus, Bacillus thuringiensis and Bacillus anthracis extracellular proteomes Proteomics 2005 5 3696 3711 16167365 10.1002/pmic.200401225

[16] Kalkum M Lyon GJ Chait BT Detection of secreted peptides by using hypothesis-driven multistage mass spectrometry Proc. Natl. Acad. Sci. USA 2003 100 2795 2800 12591958 10.1073/pnas.0436605100 PMC151420

[17] Saier MH Jr Protein secretion and membrane insertion systems in gram-negative bacteria J. Membr. Biol 2006 214 75 90 17546510 10.1007/s00232-006-0049-7

[18] Choo KH Tan TW Ranganathan S A comprehensive assessment of *N-*terminal signal peptides prediction methods BMC Bioinformatics 2009 10 S2 19958512 10.1186/1471-2105-10-S15-S2 PMC2788353

[19] Bendtsen JD Nielsen H von Heijne G Brunak S Improved prediction of signal peptides: SignalP 3.0 J. Mol. Biol 2004 340 783 795 15223320 10.1016/j.jmb.2004.05.028

[20] Emanuelsson O Brunak S von Heijne G Nielsen H Locating proteins in the cell using TargetP, SignalP and related tools Nat. Protoc 2007 2 953 971 17446895 10.1038/nprot.2007.131

[21] Erickson BK Mueller R Verberkmoes NC Shah M Singer SW Thelen M Banfield JF Hettich RL Computational Prediction and Experimental Validation of Signal Peptide Cleavages in the Extracellular Proteome of a Natural Microbial Community J. Proteome Res 2010 9 2148 2159 20218729 10.1021/pr900877a

[22] Leversen NA de Souza GA Malen H Prasad S Jonassen I Wiker HG Evaluation of signal peptide prediction algorithms for identification of mycobacterial signal peptides using sequence data from proteomic methods Microbiology 2009 155 2375 2383 19389770 10.1099/mic.0.025270-0 PMC2885676

[23] Zhou M Boekhorst J Francke C Siezen R LocateP: Genome-scale subcellular-location predictor for bacterial proteins BMC Bioinformatics 2008 9 173 18371216 10.1186/1471-2105-9-173 PMC2375117

[24] Josic D Kovac S Application of proteomics in biotechnology—microbial proteomics Biotechnol. J 2008 3 496 509 18320565 10.1002/biot.200700234

[25] Keller M Hettich R Environmental proteomics: a paradigm shift in characterizing microbial activities at the molecular level Microbiol. Mol. Biol. Rev 2009 73 62 70 19258533 10.1128/MMBR.00028-08 PMC2650886

[26] Schneider T Riedel K Environmental proteomics: analysis of structure and function of microbial communities Proteomics 2010 10 785 798 19953545 10.1002/pmic.200900450

[27] Armengaud J Proteogenomics and systems biology: quest for the ultimate missing parts Expert Rev. Proteomics 2010 7 65 77 20121477 10.1586/epr.09.104

[28] Dumas E Desvaux M Chambon C Hébraud M Insight into the core and variant exoproteomes of Listeria monocytogenes species by comparative subproteomic analysis Proteomics 2009 9 3136 3155 19526548 10.1002/pmic.200800765

[29] Westers L Westers H Zanen G Antelmann H Hecker M Noone D Devine KM van Dijl JM Quax WJ Genetic or chemical protease inhibition causes significant changes in the *Bacillus subtilis* exoproteome Proteomics 2008 8 2704 2713 18546160 10.1002/pmic.200800009

[30] Shah P Atwood JA Orlando R El Mubarek H Podila GK Davis MR Comparative proteomic analysis of *Botrytis cinerea* secretome J. Proteome Res 2009 8 1123 1130 19140674 10.1021/pr8003002

[31] Tunica D Yin X Sidibe A Stegemann C Nissum M Zeng L Brunet M Mayr M Proteomic analysis of the secretome of human umbilical vein endothelial cells using a combination of free-flow electrophoresis and nanoflow LC-MS/MS Proteomics 2009 9 4991 4996 19810032 10.1002/pmic.200900065

[32] Evans FF Raftery MJ Egan S Kjelleberg S Profiling the secretome of the marine bacterium *Pseudoalteromonas tunicata* using amine-specific isobaric tagging (iTRAQ) J. Proteome Res 2007 6 967 975 17330939 10.1021/pr060416x

[33] Liu H Sadygov RG Yates JR III A model for random sampling and estimation of relative protein abundance in shotgun proteomics Anal. Chem 2004 76 4193 4201 15253663 10.1021/ac0498563

[34] Mueller LN Brusniak M-Y Mani DR Aebersold R An assessment of software solutions for the analysis of mass spectrometry based quantitative proteomics data J. Proteome Res 2008 7 51 61 18173218 10.1021/pr700758r

[35] Kuhnert P Heyberger-Meyer B Nicolet J Frey J Characterization of PaxA and its operon: a cohemolytic RTX toxin determinant from pathogenic *Pasteurella aerogenes* Infect. Immun 2000 68 6 12 10603361 10.1128/iai.68.1.6-12.2000 PMC97094

[36] Rodriguez J Gupta N Smith RD Pevzner PA Does trypsin cut before proline? J. Proteome Res 2008 7 300 305 18067249 10.1021/pr0705035

[37] Armengaud J A perfect genome annotation is within reach with the proteomics and genomics alliance Curr. Opin. Microbiol 2009 12 292 300 19410500 10.1016/j.mib.2009.03.005

[38] Bradshaw RA Brickey WW Walker KW *N-*terminal processing: the methionine aminopeptidase and N alpha-acetyl transferase families Trends Biochem. Sci 1998 23 263 267 9697417 10.1016/s0968-0004(98)01227-4

[39] Frottin F Martinez A Peynot P Mitra S Holz RC Giglione C Meinnel T The proteomics of *N-*terminal methionine cleavage Mol. Cell. Proteomics 2006 5 2336 2349 16963780 10.1074/mcp.M600225-MCP200

[40] Giglione C Vallon O Meinnel T Control of protein life-span by *N-*terminal methionine excision EMBO J 2003 22 13 23 12505980 10.1093/emboj/cdg007 PMC140049

[41] Yeats C Bentley S Bateman A New knowledge from old: *in silico* discovery of novel protein domains in *Streptomyces coelicolor* BMC Microbiol 2003 3 3 12625841 10.1186/1471-2180-3-3 PMC151604

[42] Lally ET Hill RB Kieba IR Korostoff J The interaction between RTX toxins and target cells Trends Microbiol 1999 7 356 361 10470043 10.1016/s0966-842x(99)01530-9

[43] Li L Rock JL Nelson DR Identification and characterization of a repeat-in-toxin gene cluster in *Vibrio anguillarum* Infect. Immun 2008 76 2620 2632 18378637 10.1128/IAI.01308-07 PMC2423100

[44] Lin W Fullner KJ Clayton R Sexton JA Rogers MB Calia KE Calderwood SB Fraser C Mekalanos JJ Identification of a *Vibrio cholerae* RTX toxin gene cluster that is tightly linked to the cholera toxin prophage Proc. Natl. Acad. Sci. USA 1999 96 1071 1076 9927695 10.1073/pnas.96.3.1071 PMC15352

[45] Sasaki H Kawamoto E Tanaka Y Sawada T Kunita S Yagami K-i Identification and characterization of hemolysin-like proteins similar to RTX toxin in *Pasteurella pneumotropica* J. Bacteriol 2009 191 3698 3705 19363112 10.1128/JB.01527-08 PMC2681905

[46] Gogarten JP Senejani AG Zhaxybayeva O Olendzenski L Hilario E INTEINS: structure, function, and evolution Annu. Rev. Microbiol 2002 56 263 287 12142479 10.1146/annurev.micro.56.012302.160741

[47] Perler FB InBase: the intein database Nucleic Acids Res 2002 30 383 384 11752343 10.1093/nar/30.1.383 PMC99080

[48] Persson OP Pinhassi J Riemann L Marklund B-I Rhen M Normark S González JM Hagström Å High abundance of virulence gene homologues in marine bacteria Environ. Microbiol 2009 11 1348 1357 19207573 10.1111/j.1462-2920.2008.01861.x PMC2702493

[49] Moran MA Belas R Schell MA Gonzalez JM Sun F Sun S Binder BJ Edmonds J Ye W Orcutt B Howard EC Meile C Palefsky W Goesmann A Ren Q Paulsen I Ulrich LE Thompson LS Saunders E Buchan A Ecological genomics of marine Roseobacters Appl. Environ. Microbiol 2007 73 4559 4569 17526795 10.1128/AEM.02580-06 PMC1932822

[50] Baudet M Ortet P Gaillard J-C Fernandez B Guérin P Enjalbal C Subra G de Groot A Barakat M Dedieu A Armengaud J Proteomics-based refinement of *Deinococcus deserti* genome annotation reveals an unwonted use of non-canonical translation initiation codons Mol. Cell. Proteomics 2010 9 415 426 19875382 10.1074/mcp.M900359-MCP200 PMC2830850

[51] Stancik LM Stancik DM Schmidt B Barnhart DM Yoncheva YN Slonczewski JL pH-dependent expression of periplasmic proteins and amino acid catabolism in *Escherichia coli* J. Bacteriol 2002 184 4246 4258 12107143 10.1128/JB.184.15.4246-4258.2002 PMC135203

[52] Handa N Terada T Doi-Katayama Y Hirota H Tame JRH Park S-Y Kuramitsu S Shirouzu M Yokoyama S Crystal structure of a novel polyisoprenoid-binding protein from *Thermus thermophilus* HB8 Protein Sci 2005 14 1004 1010 15741337 10.1110/ps.041183305 PMC2253440

[53] de Groot A Dulermo R Ortet P Blanchard L Guerin P Fernandez B Vacherie B Dossat C Jolivet E Siguier P Chandler M Barakat M Dedieu A Barbe V Heulin T Sommer S Achouak W Armengaud J Alliance of proteomics and genomics to unravel the specificities of Sahara bacterium Deinococcus deserti PLoS Genet 2009 5 e1000434 19370165 10.1371/journal.pgen.1000434 PMC2669436

[54] Dupierris V Masselon C Court M Kieffer-Jaquinod S Bruley C A toolbox for validation of mass spectrometry peptides identification and generation of database: IRMa Bioinformatics 2009 25 1980 1981 19420053 10.1093/bioinformatics/btp301

[55] Zivanovic Y Armengaud J Lagorce A Leplat C Guerin P Dutertre M Anthouard V Forterre P Wincker P Confalonieri F Genome analysis and genome-wide proteomics of Thermococcus gammatolerans, the most radioresistant organism known amongst the Archaea Genome Biol 2009 10 R70 19558674 10.1186/gb-2009-10-6-r70 PMC2718504

[56] Nogueira T Rankin DJ Touchon M Taddei F Brown SP Rocha EPC Horizontal gene transfer of the secretome drives the evolution of bacterial cooperation and virulence Curr. Biol 2009 19 1683 1691 19800234 10.1016/j.cub.2009.08.056 PMC2773837

